# HTRA1, an age‐related macular degeneration protease, processes extracellular matrix proteins EFEMP1 and TSP1

**DOI:** 10.1111/acel.12710

**Published:** 2018-05-05

**Authors:** Michael K. Lin, Jin Yang, Chun Wei Hsu, Anuradha Gore, Alexander G. Bassuk, Lewis M. Brown, Ryan Colligan, Jesse D. Sengillo, Vinit B. Mahajan, Stephen H. Tsang

**Affiliations:** ^1^ College of Physicians & Surgeons Columbia University New York NY USA; ^2^ Edward S. Harkness Eye Institute New York‐Presbyterian Hospital New York NY USA; ^3^ Jonas Children's Vision Care, and Bernard & Shirlee Brown Glaucoma Laboratory Columbia Stem Cell Initiative Departments of Ophthalmology, Pathology & Cell Biology Institute of Human Nutrition Herbert Irving Comprehensive Cancer Center Columbia University New York NY USA; ^4^ Tianjin Medical University Eye Hospital Tianjin China; ^5^ Omics Laboratory Department of Ophthalmology Byers Eye Institute Stanford University Palo Alto CA USA; ^6^ Department of Pediatrics and Neurology University of Iowa Iowa City IA USA; ^7^ Quantitative Proteomics and Metabolomics Center Department of Biological Sciences Columbia University New York NY USA; ^8^ Palo Alto Veterans Administration Palo Alto CA USA

**Keywords:** age related macular degeneration, genetics, mass spectrometry, Neurodegenerative diseases

## Abstract

High‐temperature requirement protein A1 (HTRA1) is a serine protease secreted by a number of tissues including retinal pigment epithelium (RPE). A promoter variant of the gene encoding HTRA1 is part of a mutant allele that causes increased HTRA1 expression and contributed to age‐related macular degeneration (AMD) in genomewide association studies. AMD is characterized by pathological development of drusen, extracellular deposits of proteins and lipids on the basal side of RPE. The molecular pathogenesis of AMD is not well understood, and understanding dysregulation of the extracellular matrix may be key. We assess the high‐risk genotype at 10q26 by proteomic comparison of protein levels of RPE cells with and without the mutation. We show HTRA1 protein level is increased in high‐risk RPE cells along with several extracellular matrix proteins, including known HTRA1 cleavage targets LTBP‐1 and clusterin. In addition, two novel targets of HTRA1 have been identified: EFEMP1, an extracellular matrix protein mutated in Doyne honeycomb retinal dystrophy, a genetic eye disease similar to AMD, and thrombospondin 1 (TSP1), an inhibitor of angiogenesis. Our data support the role of RPE extracellular deposition with potential effects in compromised barrier to neovascularization in exudative AMD.

## 1 INTRODUCTION

Age‐related diseases, including age‐related macular degeneration (AMD), are an enormous burden and a growing public health concern, given the aging population. Molecular pathways underlying AMD have yet to be defined, mainly for two reasons: (i) studies use inappropriate cells. Most use either immature cell types (e.g., immortal cell lines) or cells derived postmortem from patients with late‐stage disease. Neither kind of cell can model the important early stages of an age‐related disease, and the latter are in limited supply and thus not amenable to high‐throughput applications. Furthermore, neither can be used to study the function of a single gene in isolation. (ii) Most age‐related diseases are not amenable to study because they have multiple genetic risk factors, the underlying cellular pathophysiology is poorly understood, and the affected cells are physically inaccessible and not easily studied in vitro.

Retinal pigment epithelial (RPE) dysfunction in age‐related macular degeneration (AMD) leads to central vision loss. The early stages of AMD are characterized by yellow deposits of drusen outside of dysfunctional RPE. Increasing size and number of drusen and the associated inflammation eventually lead to retinal degeneration as patient's age. Advanced disease is characterized by either death of macular tissue in general (termed geographic atrophy or dry AMD) or choroidal neovascularization that causes fluid to leak into the macula (termed wet AMD).

Recently, key inroads into finding the cause of AMD have been furnished by genomewide association studies (GWAS) that identified mutations at two genetic loci as risk factors for AMD.

**Figure 1:**
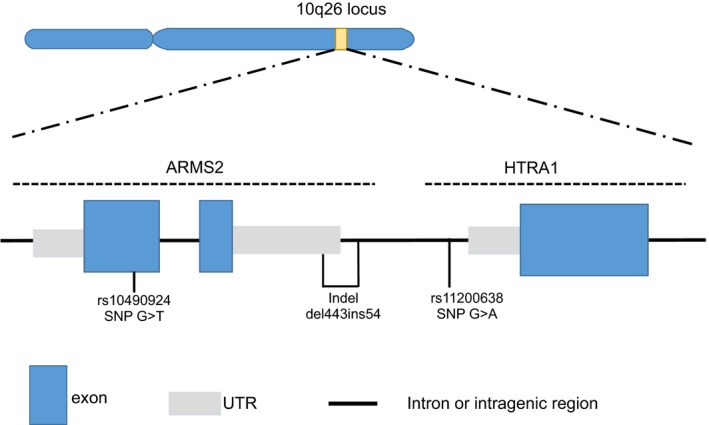
High‐risk mutations at the 10q26 locus. (a) Genomewide association studies (GWAS) link AMD to the 10q26 locus. One coding SNP (rs10490924) produces the A69S mutation in the putative *Age‐Related Maculopathy Susceptibility 2* (ARMS2) gene. ARMS2 is also affected by an insertion–deletion del443ins54 that deletes the polyadenylation signal sequences of the RNA transcript. Another SNP, rs11200638, in the promoter of *High Temperature Requirement A Serine Peptidase 1* (*HTRA1)* is also linked to AMD

An AMD‐associated genetic locus was found at 10q26, where the risk from three mutations is inseparable due to linkage disequilibrium (Figure 1). The mutations are composed of a coding SNP (rs10490924) that produces the A69S mutation in the putative *Age‐Related Maculopathy Susceptibility 2* (ARMS2) gene, an insertion–deletion del443ins54 that deletes the polyadenylation signal sequences of the RNA transcript, and the SNP, rs11200638, in the promoter of *High Temperature Requirement A Serine Peptidase 1* (*HTRA1*; Yang et al., 2006). The other major genetic locus linked to AMD is at chromosome 1q31, where a single nucleotide polymorphism (SNP) rs1061170 causes a missense mutation Y402H in complement factor H (CFH; Edwards et al., 2005; Klein et al., 2005; Hageman et al., 2005; Haines et al., 2005). These two genetic loci, 1q31 and 10q26, were the first to be identified in human GWAS, and they confer the most significant genetic risk of AMD alleles. The initial cohort studies showed the risk of developing AMD was 4.6 times that of wild type among people heterozygous and 7.4 times increased for those homozygous for the CFH risk allele (Klein et al., 2005). Those heterozygous for the ARMS2/HTRA1 risk allele were at ~2.7 times greater risk of AMD, whereas homozygotes had 8.2 times increased risk (Rivera et al., 2005). Interestingly, a retinal degenerative disease similar to AMD called Doyne honeycomb macular dystrophy was recently discovered to be caused by EFEMP1 coding mutations, and families carrying disease mutations develop drusen and retinal degeneration decades earlier than patients with AMD (Stone et al., 1999). Although there is phenotypic overlap, whether the two diseases are mechanistically linked is not known. Further molecular studies of these genetic risk factors might provide great insight into the mechanisms of these diseases.

How the HTRA1/ARMS2 risk allele at 10q26 causes AMD remains unclear, but increasing evidence suggests the gene products may also be involved in drusen formation and inflammation in the extracellular space (Iejima, Nakayama & Iwata, 2015; Jones et al., 2011). Whereas the biological relevance of the putative ARMS2 protein is not well understood, more is known about HTRA1. HTRA1 mutations also cause the genetic vascular disease, cerebral autosomal recessive arteriopathy with subcortical infarcts and leukoencephalopathy (CARASIL; Hara et al., 2009). The protein is implicated as a tumor suppressor and shown to play an important role in extracellular matrix remodeling during placental development and arthritis (Chien et al., 2004; Grau et al., 2006; Nie et al., 2006). It cleaves and inhibits TGF beta, and several extracellular matrix proteins, including fibronectin and amyloid precursor protein (Beaufort et al., 2014; Grau et al., 2005; Oka et al., 2004). Also, when HTRA1 is overexpressed in mice, the elastic network of extracellular matrix becomes fragmented between the RPE and the choroid (Vierkotten, Muether & Fauser, 2011).

We have overcome some of the hurdles to studying AMD pathogenesis by developing a method for creating patient‐derived RPE. To determine how the *HTRA1/ARMS2* risk allele at 10q26 causes AMD, we make use of new technologies in stem cells and protein biology. RPE is generated from patients' own skin cells through induced pluripotent stem (iPS) cells, yielding matched RPE that expresses key molecular markers (Liao et al., 2010). This allows us to study patient‐specific genomes. Our previous study used iPS‐RPE derived from an AMD patient with the high‐risk *HTRA1/ARMS2* allele and demonstrated decreased SOD2 activity in high‐risk RPE, implying oxidative stress might drive AMD pathogenesis (Yang et al., 2014). To provide further mechanistic insight at the functional level, we compared wild‐type RPE to that derived from an AMD patient with the high‐risk *HTRA1*/*ARMS2* allele, in a high‐throughput, mass spectrometry experiment. As expected, mass spectrometry detected increased HTRA1 expression in the high‐risk RPE cells. Surprisingly, we also found increased expression of several components of extracellular matrix proteins that HTRA1 is known to cleave and decreased expression of RNA processing proteins. Our study also identifies EFEMP1 as a cleavage target of HTRA1 implicating a potential link between the two diseases AMD and DHRD.

## 2 RESULTS

### 2.1 Differential expression of proteins in RPE cells carrying the AMD risk allele at 10q26

**Figure 2:**
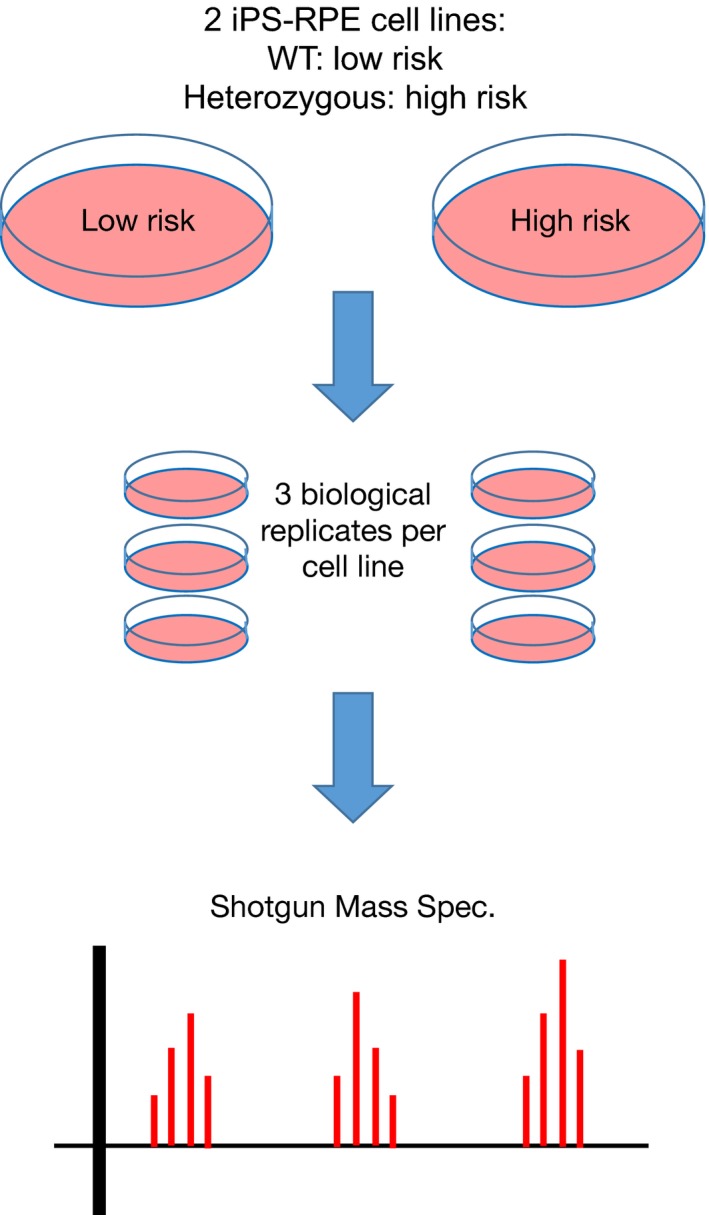
iPS‐RPE cell line experimental design: WT vs. heterozygous. iPSC‐derived RPE cell lines were created from subjects carrying both the homozygous wild‐type (low risk) and heterozygous alleles (high risk) at the AMD risk‐associated 10q26 locus. For both cell lines, three biological replicates were prepared from three separate cultures. Protein was extracted, and expression was measured using mass spectrometry‐based proteomic analysis

To assess the functional significance of the high‐risk allele composed of mutations in HTRA1/ARMS2 at 10q26, RPE from an AMD patient heterozygous (T‐in/del‐A; G‐wt‐G) at the 10q26 locus and a low‐risk homozygote (G‐wt‐G; G‐wt‐G) control at 10q26 locus was compared. An unbiased proteomic analysis compared cell lines derived from their iPS‐RPE. Cell cultures were subjected to trypsin digestion of the proteins, and label‐free, LC‐MS/MS analysis of the resulting peptides (see diagram of the experimental design in Figure 2). The expression level changed by twofold or more for 135 proteins: high‐risk cells decreased expression of 74 proteins and increased expression of 61 proteins (Table S1). Differentially expressed proteins were analyzed bioinformatically using the PANTHER GO‐Slim Overrepresentation Test.

The 74 proteins with decreased expression in high‐risk cells were found from PANTHER GO‐Slim testing to have overrepresentation of three Molecular Function terms, “mRNA binding” (13.34‐fold; *p* = .0433), “RNA binding” (9.07‐fold; *p* = 5.34 × 10^−4^), and “structural constituent of ribosome” (8.34‐fold; *p* = .0156). Three Biological Function terms were overrepresented: “mRNA splicing, via spliceosome” (13.03‐fold; *p* = 5.01 × 10^−5^), “RNA splicing, via transesterification reactions” (10.57‐fold; *p* = .0287), and “nitrogen compound metabolic process” (2.58‐fold; *p* = .0339). Two Cellular Component terms were overrepresented: “ribonucleoprotein complex” (10.5‐fold; *p* = 6.25 × 10^−5^) and “intracellular” (2.49‐fold; *p* = .0201). Over half of the proteins are localized in the nucleus and interact directly with nucleic acids, and they are shown in Table S1, categorized by gene ontology data. Proteins involved in RNA splicing were one of the largest groups found from PANTHER GO‐SLIM testing, and these proteins are listed in Table 1.

**Table 1:** Proteins decreased in high‐risk RPE cells involved in RNA splicing

P62306	SNRPF	Small nuclear ribonucleoprotein F
P23246	SFPQ	Splicing factor, proline‐ and glutamine‐rich
C9JAB2	SRSF7	Serine/arginine‐rich‐splicing factor 7
P14866	HNRNPLL	Heterogeneous nuclear ribonucleoprotein L
P52597	HNRNPF	Heterogeneous nuclear ribonucleoprotein F
P31943	HNRNPH11	Heterogeneous nuclear ribonucleoprotein H
P31942	HNRNPH3	Heterogeneous nuclear ribonucleoprotein H3
Q08211	DHX9	ATP‐dependent RNA helicase A
P38159	RBMX	RNA‐binding motif protein, X chromosome
P17844	DDX5	Probable ATP‐dependent RNA helicase DEAD box protein
P26599	PTBP1	Polypyrimidine tract‐binding protein 1
O75533	SF3B1	Splicing factor 3B subunit 1

### 2.2 RPE with the heterozygous (T‐in/del‐A; G‐wt‐G) risk 10q26 allele shows increased expression of HTRA1 and extracellular matrix proteins

The 61 proteins with increased expression in high‐risk cells were found to have overrepresentation of the gene ontology terms “extracellular matrix structural constituent” (41‐fold, *p* = 5.60 × 10^−4^), “actin binding” (8.98‐fold; *p* = .0437), “structural constituent of cytoskeleton” (5.35‐fold; *p* = 2.87 × 10^−3^), and “structural molecule activity” (4.8‐fold; 1.67 × 10^−4^) in the Molecular Function classification; “protein folding” (12.22; *p* = .0144) in the Biological Function classification; and “extracellular matrix” (8.59‐fold; *p* = .0183) in the Cellular Component classification. Actin‐binding proteins were predominantly increased in cells from high‐risk AMD at the 10q26 locus, along with many extracellular matrix proteins, especially proteins related to fibrillin microfibrils (Table S2). The largest gene ontology group increased in RPE cells with the AMD risk allele at 10q26 was extracellular matrix proteins, which are listed in Table 2.

**Table 2:** Proteins increased in high‐risk RPE cells localized in the extracellular matrix

Q92743	HTRA1	Serine protease HTRA1
P35555	FBN1	Fibrillin‐1
P35556	FBN2	Fibrillin‐2
Q14766	LTBP1	Latent‐transforming growth factor beta‐binding protein 1
Q14767	LTBP2	Latent‐transforming growth factor beta‐binding protein 2
Q12805	EFEMP1	EGF‐containing fibulin‐like extracellular matrix protein 1 (fibulin 3)
O95967	EFEMP2	EGF‐containing fibulin‐like extracellular matrix protein 2 (fibulin 4)
P10909	CLU	Clusterin
P02462	COL4A1	Collagen alpha‐1(IV) chain
P08572	COL4A2	Collagen alpha‐2(IV) chain
P07996	THBS1	Thrombospondin‐1
O15230	LAMA5	Laminin subunit alpha‐5
P55083	MFAP4	Microfibril‐associated glycoprotein 4
P13611	VCAN	Versican core protein
P69905	HBA1	Hemoglobin subunit alpha
Q08431	MFGE8	Lactadherin
P16278	GLB1	Beta‐galactosidase
PRSS33	PRSS33	Serine protease 33

High‐risk RPE cells contained more HTRA1, providing support that the high‐risk allele at 10q26 causes HTRA1 to be overexpressed. Also upregulated were several extracellular matrix proteins, including the HTRA1 cleavage targets, LTBP1 and clusterin. From this novel finding, we postulated that HTRA1 cleavage of these proteins might be related to their increased abundance.

**Figure 3:**
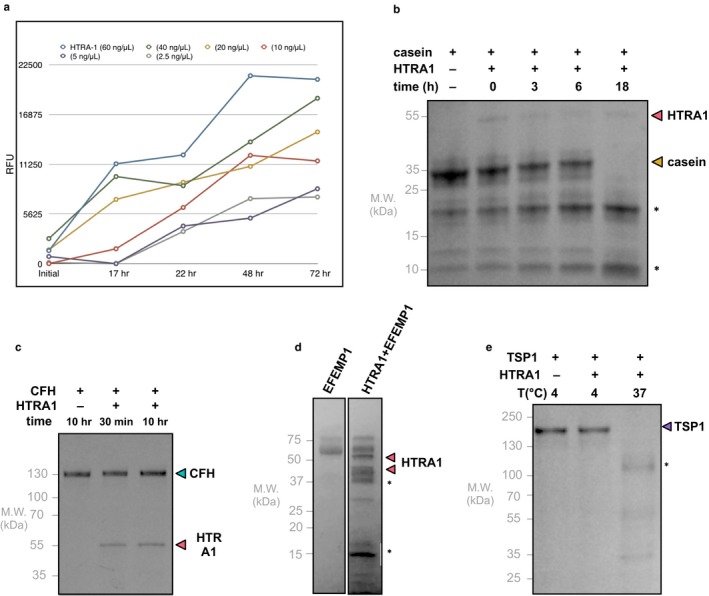
HTRA1 cleaves target substrates. (a) The enzymatic activity of recombinant HTRA1 was confirmed using a protease activity kit. The cleavage of HTRA1 β‐casein was measured and is shown to increase as both the time period and concentration of HTRA1 are increased. (b) Casein, a known HTRA1 substrate, is cleaved by recombinant HTRA1. (c) CFH is not cleaved by recombinant HTRA1, validating previous reports which show that it is not an HTRA1 target. (d) After a 90‐min incubation, recombinant EFEMP1 is partially cleaved by recombinant HTRA1 and is fully cleaved following an overnight incubation. (e) Recombinant TSP1 is also fully cleaved by recombinant HTRA1 after an overnight incubation

First, the enzymatic activity of the recombinant HTRA1 was assessed using a protease activity kit. Using beta‐casein, a known HTRA1 substrate, cleavage of casein by HTRA1 was measured at different HTRA1 concentrations and incubation times. Cleavage of casein was measured by monitoring fluorescence intensity. We found that HTRA1 cleavage of substrates increases proportionally to both the concentration of HTRA1 and incubation time (Figure 3a). Next, we validated HTRA1 cleavage of two candidate targets found in the extracellular matrix that have established roles in retinal degenerative disease—EFEMP1 and TSP1. In vitro cleavage assays used recombinant proteins, and casein (a known HTRA1 substrate) as the positive control for HTRA1 cleavage (Figure 3b). Our negative control was WT CFH, which previous reports showed is not cleaved by HTRA1 (Figure 3c). In these assays, recombinant HTRA1 cleaved recombinant EFEMP1, partially after a 90‐min incubation and completely after an overnight incubation (Figure 3d). TSP1 was also cleaved after an overnight incubation (Figure 3e).

**Figure 4:**
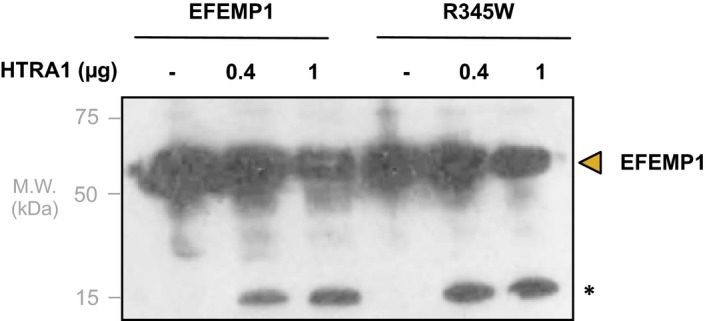
Recombinant HTRA1 cleavage of native EFEMP and EFEMP1, R345W. Overnight incubation of recombinant HTRA1 and native EFEMP1 shows a diminished EFEMP1 band as well as a cleavage product at 15 kDa. This cleavage pattern is unaltered when recombinant HTRA1 is instead incubated with p.R345W EFEMP1 (Doyne macular dystrophy mutation)

Next, we examined whether recombinant HTRA1 cleaved native EFEMP1. Human primary fibroblast cells were cultured and used to prepare protein extracts to which recombinant HTRA1 was added. Cleavage assays were immunoblotted with monoclonal anti‐EFEMP1 antibodies, showing a diminished signal for native EFEMP1 and generation of a 15 kD cleavage fragment, suggesting HTRA1 cleaved EFEMP1 (Figure 4).

Doyne honeycomb macular dystrophy (Malattia Leventinese, OMIM 126600), a retinal degenerative disorder similar to AMD, is caused by the p.R345W mutation in *EFEMP1*. We tested whether this mutation would affect HTRA1 cleavage. As before, we cultured patient fibroblasts and used them to prepare lysates for cleavage assays. In this experiment, the p.R345W EFEMP1 in Doyne patient lysates incubated with HTRA1 and compared to WT EFEMP1 (Figure 4). As shown in the immunoblot, there was no difference in HTRA1 cleavage of WT and p.R345W mutant EFEMP1.

## 3 DISCUSSION

Four DNA variants that are strong AMD risk factors were recently identified in genomewide association studies (GWAS). Three of these variants are located in the tightly linked *HTRA1* and *ARMS2* genes. Such physical genomic proximity means that they will always co‐segregate during inheritance and cannot be analyzed independently. The high‐risk‐conferring allele is noncoding for *HTRA1*, which has presented a challenge for investigators to determine its biological relevance, and there is controversial evidence the mutant allele causes increased HTRA1 protein levels. Studying the molecular underpinnings of AMD has also been hindered by other important experimental limitations, which include lack of a rodent model, difficulty obtaining primary tissue given that human RPE can only be obtained via autopsy or enucleation, and tissue culture models that do not express key RPE markers. To circumvent these issues, patient‐specific stem cell modeling offers the potential to differentiate pluripotent cells into RPE that manifest molecular and biological traits of in vivo disease pathology. Induced pluripotent stem (iPS) cells offer an additional advantage to study specific genetic risk alleles from afflicted individuals. In this study, we used untargeted proteomics to explore the impact of the mutant allele of the 10q26 locus. We used label‐free mass spectrometry to compare the protein expression between iPS‐RPE derived from a human subject with a heterozygous mutant allele and a human subject wild type at the 10q26 locus.

Our results showed increased expression of HTRA1 in the high‐risk RPE cells, but we did not detect the ARMS2 protein, perhaps due to low expression levels. Interestingly there was a greater than twofold increase in the expression of many actin‐binding proteins along with key components of fibrillin microfibrils in the extracellular matrix including fibrillin 1, fibrillin 2, LTBP1, LTBP2, EFEMP1, EFEMP2, and TSP1. Histologically, patients with early stages of AMD are characterized by the presence of drusen, extracellular debris that accumulates between the retinal pigment epithelium (RPE) and Bruch's membrane. Studies of the composition of drusen revealed extracellular matrix proteins as well as inflammatory molecules as the major components of drusen (Crabb et al., 2002; Wang et al., 2010). Many of the extracellular matrix proteins with increased expression in AMD high‐risk RPE cells from our data were also found in proteomic analyses of drusen. A possible reason for the increase in extracellular matrix proteins found in heterozygous 10q26 RPE is that increased HTRA1 cleavage activity created extracellular debris in the sub‐RPE space similar to basal lamina deposits that were not cleared, but instead lead to a feedback loop to produce more of the HTRA1 cleavage substrates. Our method detected both intracellular and extracellular proteins, and future experiments to localize the increased protein levels would be informative. Further studies are needed to demonstrate whether the increased protein expression is reproducible with other human subjects carrying the AMD high‐risk allele at 10q26, whether there might be further enhancement of the differential protein expression with a homozygous mutant allele, and whether the increased expression of extracellular matrix proteins might be linked to drusen formation and other mechanistic events in AMD disease progression.

For example, GWAS data also revealed that patients homozygous for both the CFH and ARMS2/HTRA1 risk alleles had over 50 times the risk of developing AMD (Schaumberg, Hankinson, Guo, Rimm & Hunter, 2007). Despite the well‐established disease risk conferred by variants at the genetic loci of CFH and HTRA1/ARMS2, the mechanisms linking these gene products to disease are not well understood. Nevertheless, this association between CFH and AMD implicated the alternative complement pathway in AMD pathogenesis. Complement activation is normally inhibited when CFH binds C3b and Complement Reactive Protein (CRP). Thus, if the CFH Y402H risk mutation lowers its affinity for CRP, then the lack of inhibition might be expected to trigger overactive inflammation in the extracellular space. The CFH risk allele is associated with increased soft drusen in the eye, and immunohistochemical and proteomic studies of drusen composition revealed several complement proteins in addition to CFH (Crabb et al., 2002; Magnusson et al., 2005; Wang et al., 2010). Several clinical trials have tried using anti‐inflammatory agents to target the complement system and systemic inflammation, but these had limited success (Ambati, Atkinson & Gelfand, 2013).

In addition to the extracellular matrix proteins found to be increased in AMD high‐risk RPE cells, there was decreased expression of RNA splicing proteins. These proteins have not been observed before in relation to retinal degenerative disease, and while it is possible that other, unidentified, genetic differences between the two cell lines could explain the abundance difference in RNA processing proteins, their importance remains to be identified.

**Figure 5:**
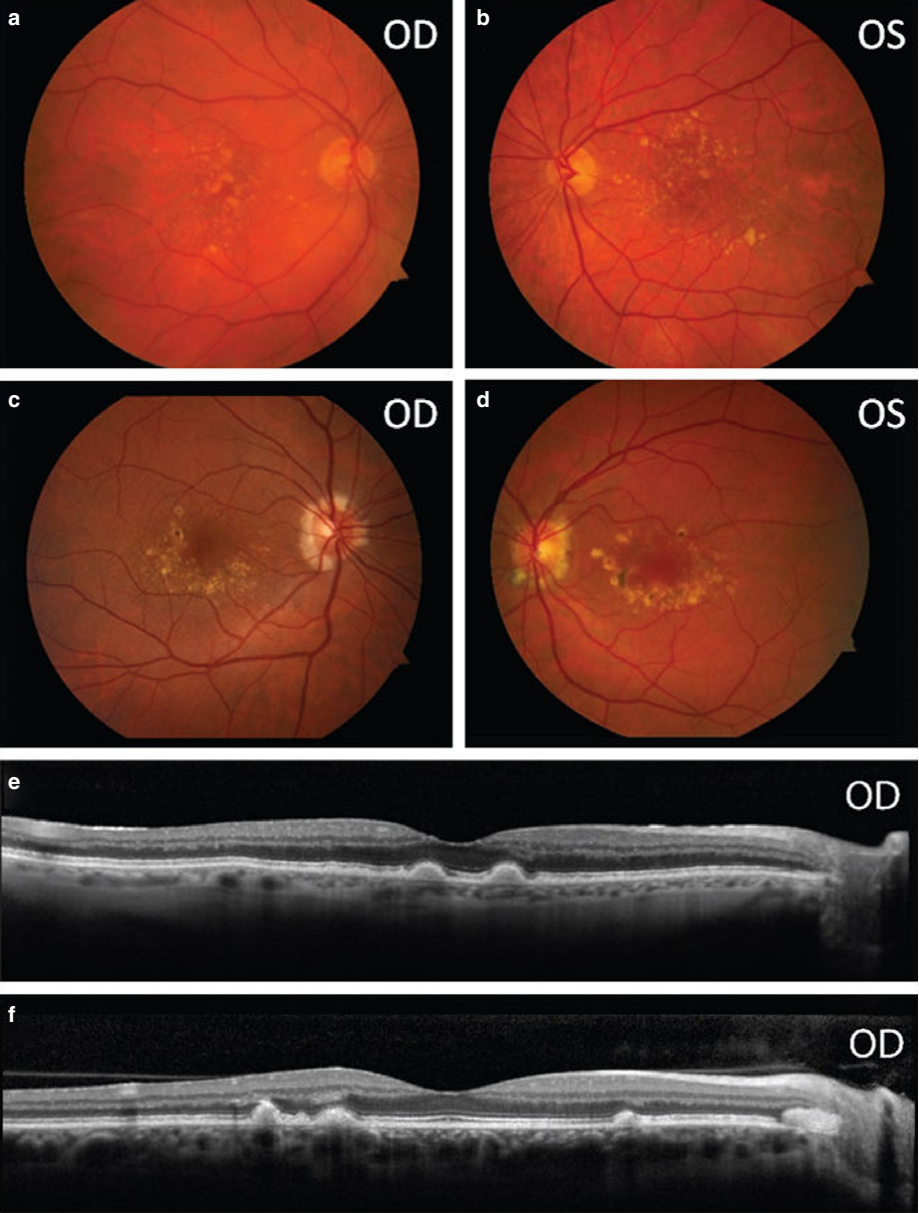
Phenotypic overlap of age‐related macular degeneration with ARMS2 and CFH risk alleles and Doyne honeycomb retinal dystrophy. Color fundus photographs of an AMD patient with *ARMS2 GT* and *CFH CC* risk alleles (a and b) and a patient with Doyne honeycomb retinal dystrophy (DHRD, c and d). Drusen in both diseases can appear similar on funduscopy, with DHRD having drusen around and on the nasal optic disc more frequently. High‐resolution foveal SD‐OCT scans of the right eyes of the same patients (third and fourth rows) illustrate that drusen are relatively indistinguishable between each disease when viewed in cross section, with marked disruption of retinal laminae in AMD and DHRD

We identified two novel substrates of the HTRA1 protease, EFEMP1 and TSP1. EFEMP1 is an extracellular matrix protein that is a component of fibrillin microfibrils, which are important both for maintaining the structure of the extracellular matrix and for regulating TGF beta activation of angiogenesis. The R345W mutation of EFEMP1 is responsible for Doyne honeycomb retinal dystrophy (DHRD), a retinal degenerative disease clinically similar to AMD that afflicts patients decades earlier than AMD (Figure 5). Mutant EFEMP1 was shown to be misfolded and retained in the cell rather than secreted into the extracellular matrix in human cell culture (Marmorstein et al., 2002). Mice with EFEMP1 knocked‐in exhibited extracellular drusen‐like deposits between the RPE and Bruch's membrane characteristic of DHRD and AMD (Marmorstein, McLaughlin, Peachey, Sasaki & Marmorstein, 2007). The sub‐RPE deposits were found to contain EFEMP1 and to activate the complement pathway (Fu et al., 2007). Our study showed increased EFEMP1 expression due to HTRA1/ARMS2 mutation and cleavage of EFEMP1 by HTRA1, which potentially links AMD with DHRD given that in both disease models EFEMP1 levels were increased in ways possibly related to structural changes in the protein. This would imply that EFEMP1 and potentially other binding partners in the extracellular matrix might have important roles in the pathogenesis of both diseases. If the mechanistic link between AMD and DHRD can be firmly established, AMD could be better studied using the genetic animal models already existing for DHRD. Thrombospondin (TSP1) is another target of HTRA1 cleavage we found. TSP1 is a potent inhibitor of angiogenesis also localized in the extracellular matrix. Cleavage of EFEMP1 and TSP1 suggest a role of HTRA1 in regulating the extracellular matrix, and potentially playing a role in the neovascularization found in exudative AMD. Increasing size and number of drusen and the associated inflammation eventually lead to retinal degeneration as patient's age. Further studies will need to be performed to determine the precise roles of these interactions.

## 4 EXPERIMENTAL PROCEDURES

### 4.1 Research subjects

Subjects were enrolled in the study with full informed consent. The protocol was approved by the Institutional Review Board at Columbia University, and the study conformed to the tenets of the Declaration of Helsinki. Each subject received a complete ophthalmic evaluation by the corresponding author. Skin biopsies were obtained from the subjects, and the samples were processed and cultured according to published protocol (Li et al., 2012).

### 4.2 Cell culture

Primary epidermal cell cultures were established by plating skin biopsies in DMEM with 10% fetal bovine serum and 100u/ml penicillin–streptomycin, and incubated at 37°C under 5% CO2. iPSC‐derived RPE cell lines were created from subjects carrying both the homozygous wild‐type and heterozygous alleles at the AMD risk‐associated 10q26 locus as described in Yang et al. (2014). Briefly, lentiviral vectors were used to transduce human fibroblasts with OCT4, SOC2, KLF4, and MYC, and cultured in human embryonic stem cell medium with 10 mm basic fibroblast growth factor (FGF), and co‐cultured with mitomycin‐C‐treated mouse embryonic fibroblast cells. iPS colonies were differentiated with addition of 10 nm nicotinamide to the culture medium. Pigmented colonies appeared after over 6 weeks and were replated onto Matrigel (BD)‐coated plates using RPE culture medium in further experiments.

### 4.3 Mass spectrometry‐based proteomic analysis

Label‐free protein expression was assessed using a NanoAcquity liquid chromatograph and a Synapt G2 HDMS QTOF mass spectrometer (Waters Corp). For both cell lines, three biological replicates were prepared from three separate cultures. Protein extraction was performed using TRIS‐buffered saline with sodium dodecyl sulfate followed by cell scraping, and chloroform–methanol precipitation was performed before dissolving in 0.1% RapiGest surfactant (Waters 186001861) with 50 mm ammonium bicarbonate. Samples were reduced with dithiothreitol and sonicated and boiled for 5 min. Protein concentration was measured with the Bradford assay. Proteins were alkylated with iodoacetamide and digested with trypsin. 50 fmol of digested yeast alcohol dehydrogenase was added as an internal detection control. Three 120 min liquid chromatography (LC)/mass spectrometry (MS) runs were performed for each biological replicate producing chromatograms in resolution/ion mobility mode.

Spectra were analyzed with ProteinLynx Global Server V.2.5, RC9, (Waters Corp). Data were further analyzed using NCBI Refseq database of human sequences, and quantitation was performed using TransOmics software (Waters Corp). Statistical comparison protein expression of the two cell lines was performed using t tests. Criteria for significant difference between cell lines were the following: at least 2 peptides for identification and quantification, at least twofold change, and *t* test *p* value <.01. Gene ontology information for differentially expressed proteins was annotated using the PANTHER Overrepresentation Test (http://www.pantherdb.org; PANTHER version 11.1 Released 2016‐10‐24).

### 4.4 Protease cleavage assay

The enzymatic activity of recombinant HTRA1 (Abcam, 134441) was confirmed with a protease activity assay kit (Abcam, 112152) containing a fluorometric casein conjugate. 10 μl of the casein conjugate, diluted at 1:20 in the kit's included assay buffer (50 mm Tris‐HCl, pH 7.5, 150 mm NaCl, 5 mm CaCl2, 0.05% Brij–35), was added to all control and test wells of a Corning 96 Round Bottom Transparent Polystyrol plate. 10 μl of 10 ng/μl trypsin in assay buffer was added to each positive control well. 10 μl of HTRA1 in assay buffer solutions of varying concentrations was added to test wells. Each assay was repeated several times and in triplicate. The loaded 96‐well plates were kept at 45°C for their incubation period. Cleavage of fluorometric casein was measured by monitoring fluorescence intensity at Ex/Em = 490/525 nm with a Tecan Infinite 200 Pro microplate reader with Tecan i‐control software.

For the protease cleavage assays on recombinant proteins, recombinant HTRA1 5 μg/25 μl (Abcam), recombinant EFEMP1 (R&D), recombinant TSP1 (R&D), recombinant CFH (CompTech), and recombinant casein (Sigma) were reconstituted individually in tris‐buffered saline pH 7.5. Casein was used as the positive control, and CFH was the negative control. Target proteins were incubated with or without HTRA1 for either 90 min or overnight at 37°C. The reaction products were resolved using SDS‐PAGE and stained with Coomassie Brilliant Blue R‐250 (Thermo).

For the protease cleavage assays on native protein from primary cell culture, cells were lysed and protein concentrations measured using the BCA protein assay. Proteins were separated by SDS‐PAGE (4%–15%; Bio‐Rad) and transferred to nitrocellulose (Bio‐Rad). After blocking, membranes were incubated in polyclonal rabbit antifibulin 3 Ab (1:1,000; catalog 5213; Prosci) overnight at 4°C, washed, and then incubated in goat anti‐rabbit IgG‐HRP secondary Ab (1:2,000; catalog sc‐2004; Santa Cruz Biotechnology Inc.) for 1 hour at room temperature. Membranes were visualized by chemiluminescence detection (EMD Millipore) using BioMax film (Kodak).

## CONFLICT OF INTEREST

The authors declare no competing financial interests.

## References

[acel12710-bib-0001] Ambati J. , Atkinson J. P. , Gelfand B. D. (2013). Immunology of age‐related macular degeneration. Nature Reviews Immunology, 13, 438–451. 10.1038/nri3459 PMC3941009 23702979

[acel12710-bib-0002] Beaufort N. , Scharrer E. , Kremmer E. , Lux V. , Ehrmann M. , Huber R. , … Dichgans M. (2014). Cerebral small vessel disease‐related protease HtrA1 processes latent TGF‐β binding protein 1 and facilitates TGF‐β signaling. Proceedings of the National Academy of Sciences of the United States of America, 111, 16496–16501. 25369932 10.1073/pnas.1418087111 PMC4246310

[acel12710-bib-0003] Chien J. , Staub J. , Hu S. I. , Erickson‐Johnson M. R. , Couch F. J. , Smith D. I. , … Shridhar V. (2004). A candidate tumor suppressor HtrA1 is downregulated in ovarian cancer. Oncogene, 23, 1636–1644. 14716297 10.1038/sj.onc.1207271

[acel12710-bib-0004] Crabb J. W. , Miyagi M. , Gu X. , Shadrach K. , West K. A. , Sakaguchi H. , … Salomon R. G. (2002). Drusen proteome analysis: an approach to the etiology of age‐related macular degeneration. Proceedings of the National Academy of Sciences of the United States of America, 99, 14682–14687. 12391305 10.1073/pnas.222551899 PMC137479

[acel12710-bib-0005] Edwards A. O. , Ritter R. , Abel K. J. , Manning A. , Panhuysen C. , & Farrer L. A. (2005). Complement factor H polymorphism and age‐related macular degeneration. Science, 308, 421–424. 10.1126/science.1110189. 15761121

[acel12710-bib-0006] Fu L. , Garland D. , Yang Z. , Shukla D. , Rajendran A. , Pearson E. , … Pierce E. A. (2007). The R345W mutation in EFEMP1 is pathogenic and causes AMD‐like deposits in mice. Human Molecular Genetics, 16, 2411–2422. 17666404 10.1093/hmg/ddm198

[acel12710-bib-0007] Grau S. , Baldi A. , Bussani R. , Tian X. , Stefanescu R. , Przybylski M. , … Ehrmann M. (2005). Implications of the serine protease HtrA1 in amyloid precursor protein processing. Proceedings of the National Academy of Sciences of the United States of America, 102, 6021–6026. 15855271 10.1073/pnas.0501823102 PMC1087941

[acel12710-bib-0008] Grau S. , Richards P. J. , Kerr B. , Hughes C. , Caterson B. , Williams A. S. , … Ehrmann M. (2006). The role of human HtrA1 in arthritic disease. Journal of Biological Chemistry, 281, 6124–6129. 16377621 10.1074/jbc.M500361200

[acel12710-bib-0009] Hageman G. S. , Anderson D. H. , Johnson L. V. , Hancox L. S. , Taiber A. J. , Hardisty L. I. , … Smith R. J. (2005). A common haplotype in the complement regulatory gene factor H (HF1/CFH) predisposes individuals to age‐related macular degeneration. Proceedings of the National Academy of Sciences of the United States of America, 102, 7227–7232. 15870199 10.1073/pnas.0501536102 PMC1088171

[acel12710-bib-0010] Haines J. L. , Hauser M. A. , Schmidt S. , Scott W. K. , Olson L. M. , Gallins P. , … Schnetz‐Boutaud N. (2005). Complement factor H variant increases the risk of age‐related macular degeneration. Science, 308, 419–421. 15761120 10.1126/science.1110359

[acel12710-bib-0011] Hara K. , Hara K. , Shiga A. , Fukutake T. , Nozaki H. , Miyashita A. , Yokoseki A. , … Ikeda M. (2009). Association of HTRA1 mutations and familial ischemic cerebral small‐vessel disease. New England Journal of Medicine, 360, 1729–1739. 19387015 10.1056/NEJMoa0801560

[acel12710-bib-0012] Iejima D. , Nakayama M. , Iwata T. (2015). HTRA1 overexpression induces the exudative form of age‐related macular degeneration. Journal of Stem Cells, 10, 193–203. 27125063

[acel12710-bib-0013] Jones A. , Kumar S. , Zhang N. , Tong Z. , Yang J. H. , Watt C. , … Yang Z. (2011). Increased expression of multifunctional serine protease, HTRA1, in retinal pigment epithelium induces polypoidal choroidal vasculopathy in mice. Proceedings of the National Academy of Sciences of the United States of America, 108, 14578–14583. 10.1073/pnas.1102853108. 21844367 PMC3167497

[acel12710-bib-0014] Klein R. J. , Zeiss C. , Chew E. Y. , Tsai J. Y. , Sackler R. S. , Haynes C. , … Bracken M. B. (2005). Complement factor H polymorphism in age‐related macular degeneration. Science, 308, 385–389. 15761122 10.1126/science.1109557 PMC1512523

[acel12710-bib-0015] Li Y. , Tsai Y. T. , Hsu C. W. , Erol D. , Yang J. , Wu W. H. , … Tsang S. H. (2012). Long‐term safety and efficacy of human‐induced pluripotent stem cell (iPS) grafts in a preclinical model of retinitis pigmentosa. Molecular Medicine, 18, 1312. 22895806 10.2119/molmed.2012.00242 PMC3521789

[acel12710-bib-0016] Liao J.‐L. , Yu J. , Huang K. , Hu J. , Diemer T. , Ma Z. , … Bok D. (2010). Molecular signature of primary retinal pigment epithelium and stem‐cell‐derived RPE cells. Human Molecular Genetics, 19, 4229–4238. 20709808 10.1093/hmg/ddq341 PMC3115666

[acel12710-bib-0017] Magnusson K. P. , Duan S. , Sigurdsson H. , Petursson H. , Yang Z. , Zhao Y. , … Helgadottir G. (2005). CFH Y402H confers similar risk of soft drusen and both forms of advanced AMD. PLoS Medicine, 3, e5. 16300415 10.1371/journal.pmed.0030005 PMC1288033

[acel12710-bib-0018] Marmorstein L. Y. , McLaughlin P. J. , Peachey N. S. , Sasaki T. , Marmorstein A. D. (2007). Formation and progression of sub‐retinal pigment epithelium deposits in Efemp1 mutation knock‐in mice: a model for the early pathogenic course of macular degeneration. Human Molecular Genetics, 16, 2423–2432. 17664227 10.1093/hmg/ddm199

[acel12710-bib-0019] Marmorstein L. Y. , Munier F. L. , Arsenijevic Y. , Schorderet D. F. , McLaughlin P. J. , Chung D. , … Marmorstein A. D. (2002). Aberrant accumulation of EFEMP1 underlies drusen formation in Malattia Leventinese and age‐related macular degeneration. Proceedings of the National Academy of Sciences of the United States of America, 99, 13067–13072. 12242346 10.1073/pnas.202491599 PMC130587

[acel12710-bib-0020] Nie G. , Hale K. , Li Y. , Manuelpillai U. , Wallace E. M. , & Salamonsen L. A. (2006). Distinct expression and localization of serine protease HtrA1 in human endometrium and first‐trimester placenta. Developmental Dynamics, 235, 3448–3455. 17072861 10.1002/dvdy.20999

[acel12710-bib-0021] Oka C. , Tsujimoto R. , Kajikawa M. , Koshiba‐Takeuchi K. , Ina J. , Yano M. , … Matsumoto M. (2004). HtrA1 serine protease inhibits signaling mediated by Tgfβ family proteins. Development, 131, 1041–1053. 14973287 10.1242/dev.00999

[acel12710-bib-0022] Rivera A. , Fisher S. A. , Fritsche L. G. , Keilhauer C. N. , Lichtner P. , Meitinger T. , & Weber B. H. (2005). Hypothetical LOC387715 is a second major susceptibility gene for age‐related macular degeneration, contributing independently of complement factor H to disease risk. Human Molecular Genetics, 14, 3227–3236. 16174643 10.1093/hmg/ddi353

[acel12710-bib-0023] Schaumberg D. A. , Hankinson S. E. , Guo Q. , Rimm E. , Hunter D. J. (2007). A prospective study of 2 major age‐related macular degeneration susceptibility alleles and interactions with modifiable risk factors. Archives of Ophthalmology, 125, 55–62. 17210852 10.1001/archopht.125.1.55

[acel12710-bib-0024] Stone E. M. , Munier A. J. , Munier F. L. , Héon E. , Piguet B. , Guymer R. H. , … Silvestri G. (1999). A single EFEMP1 mutation associated with both Malattia Leventinese and Doyne honeycomb retinal dystrophy. Nature Genetics, 22, 199–202. 10369267 10.1038/9722

[acel12710-bib-0025] Vierkotten S. , Muether P. S. , Fauser S. (2011). Overexpression of HTRA1 leads to ultrastructural changes in the elastic layer of Bruch's membrane via cleavage of extracellular matrix components. PLoS One, 6, e22959. 21829675 10.1371/journal.pone.0022959 PMC3149070

[acel12710-bib-0026] Wang L. , Clark M. E. , Crossman D. K. , Kojima K. , Messinger J. D. , Mobley J. A. , & Curcio C. A. (2010). Abundant lipid and protein components of drusen. PLoS One, 5, e10329. 20428236 10.1371/journal.pone.0010329 PMC2859054

[acel12710-bib-0027] Yang Z. , Camp N. J. , Sun H. , Tong Z. , Gibbs D. , Cameron D. J. , … Chien J. (2006). A variant of the HTRA1 gene increases susceptibility to age‐related macular degeneration. Science, 314, 992–993. 17053109 10.1126/science.1133811

[acel12710-bib-0028] Yang, J. , Li, Y. , Chan, L. , Tsai, Y. T. , Wu, W. H. , Nguyen, H. V. , … Sparrow, J. R. (2014). Validation of GWAS alleles with patient‐specific stem cell lines. Human Molecular Genetics, 23, 3445–3455. 24497574 10.1093/hmg/ddu053 PMC4049304

