# Supplementary material for: HTRA1, an age‐related macular degeneration protease, processes extracellular matrix proteins EFEMP1 and TSP1
Source: Aging Cell. 2018 May 5;17(4):e12710. doi: 10.1111/acel.12710 (PMC6052470; doi:10.1111/acel.12710)
Supplement: Supplementary file 1 [file ACEL-17-na-s001.doc]

Supplementary Table 1:

66 proteins with increased expression in high risk RPE cells are sorted with information from gene ontology groups and pubmed searches.

| Actin Binding | | |
| --- | --- | --- |
| Q9NR12 | PDLIM7 | PDZ and LIM domain protein 7 |
| P07951 | TPM2 | Tropomyosin beta chain |
| P24844 | MYL9 | Myosin regulatory light polypeptide 9 |
| P06396 | GSN | Gelsolin |
| P21333 | FLNA | Filamin-A |
| Q08043 | ACTN3 | Alpha-actinin-3 |
| Q01995 | TAGLN | Transgelin |
| P58107 | EPPK1 | Epiplakin |
| P06756 | ITGAV | Integrin alpha-V |
| P18206 | VCL | Vinculin |
|  | | |
| Cytoskeletal proteins (not actin related) | | |
| Q13509 | TUBB3 | Tubulin beta-3 chain |
| P04350 | TUBB4A | Tubulin beta-4A chain |
|  | | |
| Extracellular Matrix Components | | |
| Q92743 | HTRA1 | Serine protease HTRA1 |
| P35555 | FBN1 | Fibrillin-1 |
| P35556 | FBN2 | Fibrillin-2 |
| Q14766 | LTBP1 | Latent-transforming growth factor beta-binding protein 1 |
| Q14767 | LTBP2 | Latent-transforming growth factor beta-binding protein 2 |
| Q12805 | EFEMP1 | EGF-containing fibulin-like extracellular matrix protein 1 (fibulin 3) |
| O95967 | EFEMP2 | EGF-containing fibulin-like extracellular matrix protein 2 (fibulin 4) |
| P10909 | CLU | Clusterin |
| P02462 | COL4A1 | Collagen alpha-1(IV) chain |
| P08572 | COL4A2 | Collagen alpha-2(IV) chain |
| P07996 | THBS1 | Thrombospondin-1 |
| O15230 | LAMA5 | Laminin subunit alpha-5 |
| P55083 | MFAP4 | Microfibril-associated glycoprotein 4 |
| P13611 | VCAN | Versican core protein |
| P69905 | HBA1 | Hemoglobin subunit alpha |
| Q08431 | MFGE8 | Lactadherin |
| P16278 | GLB1 | Beta-galactosidase |
| PRSS33 | PRSS33 | Serine protease 33 |
|  | | |
| Protein folding | | |
| P13667 | PDIA4 | Protein disulfide-isomerase A4 |
| P04792 | HSPB1 | Heat shock protein beta-1 |
| P02511 | CRYAB | Alpha-crystallin B chain |
| P62942 | FKBP1A | Peptidyl-prolyl cis-trans isomerase FKBP1A |
|  | | |
| Other Proteins | | |
| O00232 | PSMD12 | 26S proteasome non-ATPase regulatory subunit 12 |
| Q02218 | OGDH | 2-oxoglutarate dehydrogenase, mitochondrial |
| O95336 | PGLS | 6-phosphogluconolactonase |
| Q86XL3 | ANKLE2 | Ankyrin repeat and LEM domain-containing protein 2 |
| P15289 | ARSA | Arylsulfatase A |
| P54687 | BCAT1 | Branched-chain-amino-acid aminotransferase, cytosolic |
| P12277 | CKB | Creatine kinase B-type |
| Q9UHL4 | DPP7 | Dipeptidyl peptidase 2 |
| P60608 | ERVFC1-1 | Endogenous retrovirus group FC1 member 1 Env polyprotein |
| Q96HE7 | ERO1L | ERO1-like protein alpha |
| Q16270 | IGFBP7 | Insulin-like growth factor-binding protein 7 |
| P10253 | GAA | Lysosomal alpha-glucosidase |
| Q99733 | NAP1L4 | Nucleosome assembly protein 1-like 4 |
| P30044 | PRDX5 | Peroxiredoxin-5, mitochondrial |
| Q9UHG3 | PCYOX1 | Prenylcysteine oxidase 1 |
| Q8IVF2 | AHNAK2 | Protein AHNAK2 |
| Q92597 | NDRG1 | Protein NDRG1 |
| Q9Y6V0 | PCLO | Protein piccolo |
| O00764 | PDXK | Pyridoxal kinase |
| Q9BRX8 | FAM213A | Redox-regulatory protein FAM213A |
| P51513 | NOVA1 | RNA-binding protein Nova-1 |
| P20794 | MAK | Serine/threonine-protein kinase MAK |
| O75368 | SH3BGRL | SH3 domain-binding glutamic acid-rich-like protein |
| Q9C093 | SPEF2 | Sperm flagellar protein 2 |
| Q9HD45 | TN9SF3 | Transmembrane 9 superfamily member 3 |
| Q12888 | TP53BP1 | Tumor suppressor p53-binding protein 1 |
| P45974 | USP5 | Ubiquitin carboxyl-terminal hydrolase 5 |

Supplementary Table 2:

74 proteins with decreased expression in high risk RPE cells are grouped using information from gene ontology and pubmed searches.

| Ribosomal components | | | | |  |
| --- | --- | --- | --- | --- | --- |
| P38919 | EIF4A3 | | Eukaryotic initiation factor 4A-III | |  |
| Q9Y262 | EIF3L | | Eukaryotic translation initiation factor 3 subunit L | |  |
| Q9UI10 | EIF2B4 | | Translation initiation factor eIF-2B subunit delta | |  |
| P05388 | RPLP0 | | 60S acidic ribosomal protein P0 | |  |
| P62277 | RPS13 | | 40S ribosomal protein S13 | |  |
| E9PB24 | RPL28 | | 60S ribosomal protein L28 | |  |
|  | | | | |  |
| RNA Splicing | | | | |  |
| P62306 | SNRPF | | Small nuclear ribonucleoprotein F | |  |
| P23246 | SFPQ | | Splicing factor, proline- and glutamine-rich | |  |
| C9JAB2 | SRSF7 | | Serine/arginine-rich-splicing factor 7 | |  |
| P14866 | HNRNPL | | Heterogeneous nuclear ribonucleoprotein L | |  |
| P52597 | HNRNPF | | Heterogeneous nuclear ribonucleoprotein F | |  |
| P31943 | HNRNPH1 | | Heterogeneous nuclear ribonucleoprotein H | |  |
| P31942 | HNRNPH3 | | Heterogeneous nuclear ribonucleoprotein H3 | |  |
| Q08211 | DHX9 | | ATP-dependent RNA helicase A | |  |
| P38159 | RBMX | | RNA-binding motif protein, X chromosome | |  |
| P17844 | DDX5 | | Probable ATP-dependent RNA helicase DEAD box protein 5 | |  |
| P26599 | PTBP1 | | Polypyrimidine tract-binding protein 1 | |  |
| O75533 | SF3B1 | | Splicing factor 3B subunit 1 | |  |
|  | | | | |  |
| DNA repair | | | | |  |
| Q13263 | TRIM28 | | Transcription intermediary factor 1-beta | |  |
| Q68DK2 | ZFYVE26 | | Zinc finger FYVE domain-containing protein 26 | |  |
| P06748 | NPM1 | | Nucleophosmin | |  |
| P78527 | PRKDC | | DNA-dependent protein kinase catalytic subunit | |  |
| P12956 | XRCC6 | | X-ray repair cross-complementing protein 6 | |  |
| P13010 | XRCC5 | | X-ray repair cross-complementing protein 5 | |  |
|  | | | | |  |
| mRNA binding | | | | |  |
| P43243 | MATR3 | | Matrin-3 | |  |
| Q15717 | ELAV1 | | ELAV-like protein 1 | |  |
| Q8NC51 | SERBP1 | | Plasminogen activator inhibitor 1 RNA-binding protein | |  |
| B0QYK0 | EWSR1 | | Ewing sarcoma breakpoint region 1 | |  |
| P05455 | SSB | | Lupus La protein | |  |
|  | | | | |  |
| rRNA processing | | | | |  |
| O00567 | NOP56 | | Nucleolar protein 56 | |  |
| Q9Y2X3 | NOP58 | | Nucleolar protein 58 | |  |
| P22087 | FBL | | rRNA 2'-O-methyltransferase fibrillarin | |  |
|  | | | | |  |
| Nucleotide biosynthesis | | | | |  |
| P23921 | RRM1 | | Ribonucleoside-diphosphate reductase large subunit | |  |
| P21589 | NT5E | | 5'-nucleotidase | |  |
| P12268 | IMPDH2 | | Inosine-5'-monophosphate dehydrogenase 2 | |  |
| P49915 | GMPS | | GMP synthase [glutamine-hydrolyzing] | |  |
| P22234 | PAICS | | Multifunctional protein ADE2 | |  |
| O14983 | ATP2A1 | | Sarcoplasmic/endoplasmic reticulum calcium ATPase 1 | |  |
|  | | | | |  |
| Nuclear export | | | | |  |
| O14980 | XPO1 | | Exportin-1 | |  |
|  | | | | |  |
| DNA processivity | | | | |  |
| P12004 | PCNA | | Proliferating cell nuclear antigen | |  |
|  | | | | |  |
| Transcription | | | | |  |
| P53999 | SUB1 | | Activated RNA polymerase II transcriptional coactivator p15 | |  |
|  | | | | |  |
| Other proteins | | | | |  |
| Q9C0C2 | TNKS1BP1 | | 182 kDa tankyrase-1-binding protein | |  |
| F5GZS6 | SLC3A2 | | 4F2 cell-surface antigen heavy chain | |  |
| P47895 | ALDH1A3 | | Aldehyde dehydrogenase family 1 member A3 | |  |
| P53675 | CLTCL1 | | Clathrin heavy chain 2 | |  |
| P54886 | ALDH18A1 | | Delta-1-pyrroline-5-carboxylate synthase | |  |
| P37059 | EDH17B2 | | Estradiol 17-beta-dehydrogenase 2 | |  |
| P43304 | GPD2 | | Glycerol-3-phosphate dehydrogenase, mitochondrial | |  |
| Q14789 | GOLGB1 | | Golgin subfamily B member 1 | |  |
| E2QRM6 | PPA2 | | Inorganic pyrophosphatase 2, mitochondrial | |  |
| O00425 | IGF2BP3 | | Insulin-like growth factor 2 mRNA-binding protein 3 | |  |
| P05362 | ICAM1 | | Intercellular adhesion molecule 1 | |  |
| P09960 | LTA4H | | Leukotriene A-4 hydrolase | |  |
| Q86W92 | PPFIBP1 | | Liprin-beta-1 | |  |
| E7EVA0 | MAP4 | | Microtubule-associated protein | |  |
| Q9Y623 | MYH4 | | Myosin-4 | |  |
| P29966 | MARCKS | | Myristoylated alanine-rich C-kinase substrate | |  |
| P15559 | NQO1 | | NAD(P)H dehydrogenase [quinone] 1 | |  |
| G3V0I5 | NDUFV1 | | NADH dehydrogenase (Ubiquinone) flavoprotein 1, 51kDa, isoform CRA_c | |  |
| O00151 | PDLIM1 | | PDZ and LIM domain protein 1 | |  |
| Q8NBJ5 | GLT25D1 | | Procollagen galactosyltransferase 1 | |  |
| Q16647 | PTGIS | | Prostacyclin synthase | |  |
| P25787 | PSMA2 | | Proteasome subunit alpha type-2 | |  |
| Q9Y4B5 | SOGA2 | | Protein SOGA2 | |  |
| P51148 | RAB5C | | Ras-related protein Rab-5C | |  |
| P00352 | ALDH1A1 | | Retinal dehydrogenase 1 | |  |
| P02787 | TF | | Serotransferrin | |  |
| Q01082 | SPTBN1 | | Spectrin beta chain, non-erythrocytic 1 | |  |
| Q9UJZ1 | STOML2 | | Stomatin-like protein 2 | |  |
| Q9P2R7 | SUCLA2 | | Succinyl-CoA ligase [ADP-forming] subunit beta, mitochondrial | |  |
| Q8IVF5 | TIAM2 | | T-lymphoma invasion and metastasis-inducing protein 2 | |  |
| P02786 | TFRC | | Transferrin receptor protein 1 | |  |
| B2RTY4 | MYO9A | | Unconventional myosin-IXa | |  |
| Q9HD67 | MYO10 | | Unconventional myosin-X | |  |
|  | |  | |  | |
|  | |  | |  | |
|  | |  | |  | |
|  | |  | |  | |
